# Online Condition Monitoring of Rotating Machines by Self-Powered Piezoelectric Transducer from Real-Time Experimental Investigations

**DOI:** 10.3390/s22093395

**Published:** 2022-04-28

**Authors:** Majid Khazaee, Lasse Aistrup Rosendahl, Alireza Rezania

**Affiliations:** Department of Energy Technology, Aalborg University, Pontoppidanstræde 111, 9220 Aalborg East, Denmark; mad@energy.aau.dk (M.K.); lar@energy.aau.dk (L.A.R.)

**Keywords:** piezoelectric, energy harvesting, motor, bearing, shaft-misalignment

## Abstract

This paper investigates self-powering online condition monitoring for rotating machines by the piezoelectric transducer as an energy harvester and sensor. The method is devised for real-time working motors and relies on self-powered wireless data transfer where the data comes from the piezoelectric transducer’s output. Energy harvesting by Piezoceramic is studied under real-time motor excitations, followed by power optimization schemes. The maximum power and root mean square power generation from the motor excitation are 13.43 mW/g^2^ and 5.9 mW/g^2^, which can be enough for providing autonomous wireless data transfer. The piezoelectric transducer sensitivity to the fault is experimentally investigated, showing the considerable fault sensitivity of piezoelectric transducer output to the fault. For instance, the piezoelectric transducer output under a shaft-misalignment fault is more than 200% higher than the healthy working conditions. This outcome indicates that the monitoring of rotating machines can be achieved by using a self-powered system of the piezoelectric harvesters. Finally, a discussion on the feasible self-powered online condition monitoring is presented.

## 1. Introduction

The global energy crisis and environmental pollution have made optimal machine operations vital, demanding less stoppage and repair costs. Online condition monitoring (OCM) is a tool to reach this optimal machine use [1]. A typical fault diagnosis involves analyzing current signals during transient start-up behavior, but some studies provide OCM methods under real-time operation [2]. The vibration characteristics from different practical fault types, e.g., electric motors, unbalanced, mechanical looseness, misalignment, bent shaft, broken bar, and bearing fault, have been previously investigated [3]. For an electrical motor, various electrical and mechanical faults have been reported. Many of these mechanical faults influence mechanical vibration and, therefore, can be detected by vibration or acoustic methods [2]. Broken rotor bar, unbalanced rotor, shaft looseness, shaft misalignment, bent shaft, and bearing faults are among the most frequently occurring faults [4]. These faults will change the characteristics of vibration measurements, either in the amplitude or frequency spectrum. Ugwiri et al. [5] used vibration signals in the frequency domain for detecting broken bar faults in asynchronous motors. Gangsar and Tiwari [6] employed time-frequency domain signals and support vector machines as the classifier for the multi-fault diagnosis. Kudelina et al. [7] investigated the motor’s bearing fault effect on the vibration level and showed that the vibration level would change noticeably. Supervised and unsupervised learning techniques are widely used in OCM. While the information about the fault is known in supervised learning techniques, there is no information on the fault state for unsupervised techniques [8]. Outlier analysis has been extensively used for fault detection as an unsupervised method [9]. The distance between the signal features is used for fault detection in the outlier analysis. 

However, the traditional OCM techniques require wired sensory measurements and wire data transfer, which will make the OCM’s development complex. One revolutionary solution are self-powered OCM methods, which are now accessible thanks to low power consumption due to advancements in microelectromechanical systems (MEMS) [10]. Regenerative energy harvesting recovers the waste energy from the environment and structures’ vibration to fabricate self-powered electronic devices [11]. Developing self-powered devices from sustainable energy sources has a revolutionary impact on industrial applications, as it will reduce energy consumption globally, eliminate the use of wiring, replace the battery, and place multi-sensors for structural monitoring will be easy. Consequently, the battery and wire production energy will be saved by achieving self-powered electronic devices.

For overcoming the conventional OCM problems, wireless sensor networks (WSNs) have been previously outlined [12]; however, the battery-powered WSNs suffer from a short battery lifetime [13]. Surrounding energy harvesting, an emerging technology [14], provides self-powered OCM systems [13]. Among energy harvesting technologies, Piezoelectricity has a relatively high power density and is easily integrated [15,16]. There are studies on the self-powered sensors for OCM applications, but the lack of practical fault demonstrations and a short lifetime can be seen. For instance, railway health monitoring by triboelectricity [17] suffers from a short lifetime because triboelectric works with friction and will be corroded over time. Alternatively, the bridge piezoelectric-based self-powered condition monitoring [17] and electromagnetic self-powered sensor [18] only focused on energy harvesting and did not study the fault and OCM. Moreover, the previous studies focused on infrastructures and large structures such asbridges [16] or wind turbine blades [19] and carried out structural imperfections such as crack and delamination. At the same time, there is a lack of self-powered OCM methods for high-speed rotating machines such as motors despite their widespread applicability and a high potential for energy harvesting.

Rotating machines with a motor are suitable kinetic energy sources as they inevitably vibrate during use. Moreover, the motor’s rotation speed is typically high, which is appropriate for piezoelectric vibration energy harvesting (PVEH). Khazaee et al. [18] analyzed the piezoelectric generator (PG) power generation under the water pump vibration, and the PG voltage output under a DC motor vibration which was shown to be 0.7 V [20]. The output power from a rotational motor ranged from 83.5–825 µW depending on the rotation speeds between 7 to 13.5 Hz [21]. Converting the motor’s rotational motion for PVEH is another idea that has been investigated by parametric (lengthwise) [22] and bending [23] mechanisms. Some PG designs are complicated or bulky, so integrating them into practical machines is difficult. The straightforward designs of PGs, which can be simply installed on currently operational machines, need more real-time testing. Moreover, in the PVEH literature, the studies only focus on the power output estimation, not the real-time fault detection.

Despite extensive studies in self-powered PVEH sensors [24,25,26,27], much of the literature is devoted to numerical/analytical simulations or laboratory-controlled sinusoidal input tests. These simulations and controlled vibrations can be different from the real-time working systems as real-time vibration is stochastic with variable statistical features. Moreover, the fault’s effects on the vibration signals can be unpredictable, so OCM performance must be evaluated under real-time tests. To sum up, testing the PGs’ performance on practical machines and real-time operational machine tests for OCM evaluation are unmet goals. This study tries to fill these unmet goals. The main novelty is the real-time investigations of the PG power output versus the electrical load and rotation speed under stochastic vibration and the fault effect on piezoelectric transducer output.

The study proposes a self-powered system that detects motor faults that change the vibration level or dominant frequency. The sensor and harvester are the piezoelectric transducers. The fault detection is based on the unsupervised outlier method by the Mahalanobis Distance (MD). The structure of this paper is organized as follows. Section 2 presents the methodology behind the paper and contains Section 2.1 on the OCM by the self-powered sensor and Section 2.2 on the design of the energy harvester. Section 3 presents the experimental motor setup working at various rotation speeds and faulty conditions. The piezoelectric generator model verification is presented in Section 4. Section 5 presents the results and the following discussions on the energy harvesting from the DC motor and moving toward a self-powering OCM system. Section 6 concludes the study’s main findings.

## 2. Methodology

### 2.1. Online Condition Monitoring (OCM) by the Self-Powered Sensor

The presented self-powered OCM technique is based on data analysis from the piezoelectric transducer outputs. Since the piezoelectric output depends on the vibration level and frequency content, faults that change the vibration level or the dominant frequency can be detected. Table 1 summarizes the faults and their effect on the machine vibration. Due to the shift in the frequency or the vibration level, the piezoelectric transducer output can detect their effect on the vibration characteristics.

**Figure 1:**
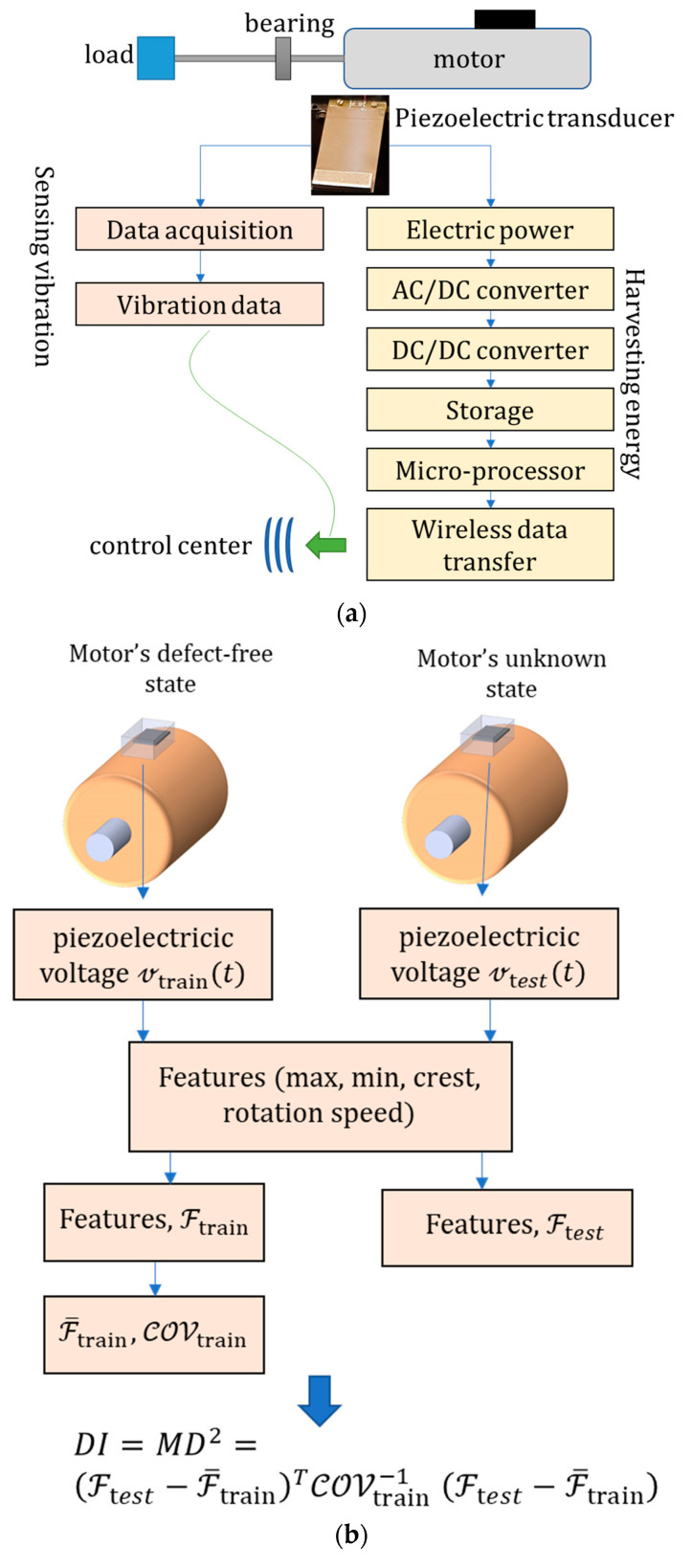
(**a**) Flowchart of the online condition monitoring by a self-powered piezoelectric transducer, and (**b**) flowchart of the fault detection by the outlier method using Mahalanobis Distance (MD).

The self-powered OCM flowchart is shown in Figure 1a. Piezoelectric serves as both sensor and energy harvesting element because of the transducing capability of piezoelectric materials and the linear mapping of vibration into a meaningful electrical signal. For the energy harvesting domain, PG generates AC power; therefore, an AC/DC converter is required, which is then connected to a DC/DC converter for regulating the voltage output. As the wireless data transfer consumes considerable energy, power storage is required to transfer the data at regular time intervals. The microprocessor regulates the data transfer intervals based on the stored electric power.

Figure 1b shows the fault detection method that is based on the outlier method using the Mahalanobis Distance (MD) function. The concept of the outlier method is that a different mechanism, probably a fault, generates observatory data that deviates considerably from the other observatory data. The MD function considers the feature covariance matrix and the average distance. The training step extracts three statistical features from piezoelectric outputs: maximum, minimum, crest factor, and one frequency feature. Since the piezoelectric output is frequency-dependent, normalized piezoelectric outputs are used. The same three features are extracted in the testing step, and the MD is calculated for each signal. The damage index is defined as the MD square value.

Let $𝓋\left(t\right)$ be the frequency normalized piezoelectric voltage with V/Hz unit, then the feature vector F is defined as:

(1) $$F={\left[max\left(𝓋\left(t\right)\right),min\left(𝓋\left(t\right)\right),Crest=\frac{max\left(𝓋\left(t\right)\right)}{min\left(𝓋\left(t\right)\right)}\right]}^{T}$$

The covariance of the features is denoted by COV. According to the training features and the covariance, the MD for an unknown machine state is defined as:

(2) $${\mathrm{MD}}^{2}={\left(F_{test}-{\bar{F}}_{train}\right)}^{T}COV_{train}^{-1}\left(F_{test}-{\bar{F}}_{train}\right)$$

By the outlier hypothesis, the unknown machine state is classified by the MD based on the following equation:

(3) $$\left\{\begin{array}{l} \mathrm{Defect}-\mathrm{free}:MD^{2}\leq \epsilon \\ \mathrm{Defected}\;:MD^{2}>\epsilon \end{array}\right.$$

wherein ϵ is the threshold.

**Figure 2:**
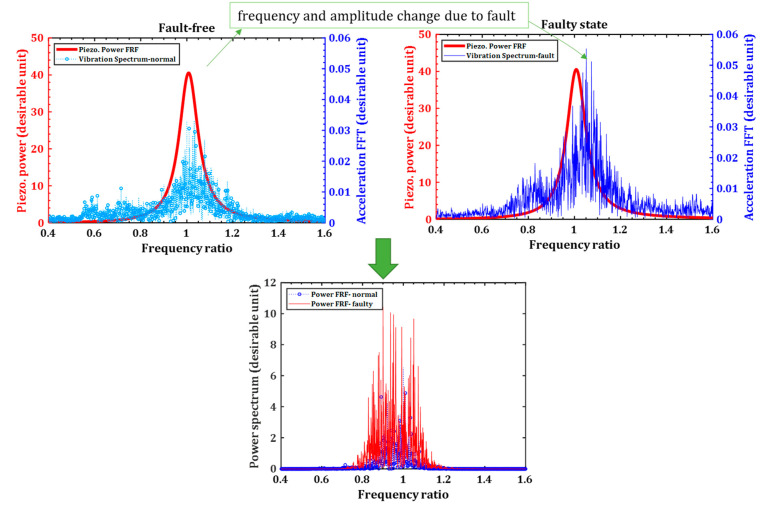
Sensitivity analysis of a piezoelectric transducer to the fault.

There is a vital need to investigate whether the piezoelectric transducer is sensitive to common motor faults. It has been shown that the motor faults change either their amplitude or frequency contents. If a PG works at a linear excitation range, e.g., low-level vibration, then the PG has a power frequency response function (FRF), showing the power generation at a specific vibration excitation. The power FRF is permanently fixed during the motor’s operation, while the acceleration signal changes due to the faults. As a result, the power output is different at different faults; thus, the piezoelectric sensor is fault-sensitive and regularly sends the PG vibration data. Figure 2 illustrates the PG fault sensitivity.

The goal is that the PG generates enough energy for the wireless data transfer and self-powers the system as well as being damage sensitive. Thus, the PG location should satisfy two goals, be sensitive to the damage, and generate enough power.

The primary bearing health is crucial for condition monitoring [29]. Moreover, many other mechanical faults can be detected from the primary bearing vibration [30]. Furthermore, the main bearing vibration is less influenced by noise and is more reliable for condition monitoring. The main bearing often emits vibration regarding energy harvesting, making it a suitable location for the PG. Therefore, an energy harvesting box is designed to be attached to the main bearing.

### 2.2. Energy Harvesting System and Piezoelectric Generator Modeling

**Figure 3:**
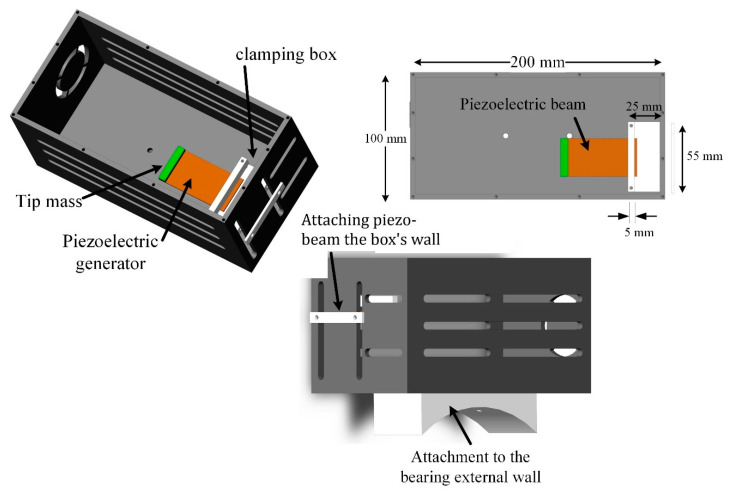
The energy harvesting box demonstrates the bearing attachment point and piezoelectric beam with the clamping box.

The energy harvesting box will be attached to the main bearing. Figure 3 demonstrates the energy harvesting box attached directly to the main bearing external wall. Because of the round shape of the external bearing wall, a circular attachment point is created. The energy harvesting box contains the slots so that multiple piezoelectric beams can be attached to the box. However, only one piezoelectric generator (PG) is attached to the box in this study. Due to the box’s complex shape and fabricating an object this lightweight, the box is fabricated using 3D printing from the plastic filament.

Moreover, a clamping box consisting of a bottom clamp base and a top clamp bar is used to create clamped-free boundary conditions for the piezoelectric energy harvesting beams. The top bar of the clamping end should have the minimum possible width to reduce the support loss damping [31]. Here, a width of 5 mm is set. Figure 3 shows the energy harvesting box and one typical attachment of one piezoelectric beam to the energy harvesting box.

Piezoceramic PZTs as high conversion materials [32] are employed. A typical piezoelectric beam consists of piezoelectric layers, substrate shim, contact layers, and added tip mass. Each of these elements has specific effects on power generation [33].

The piezoelectric output wires are directly connected to the resistive load, and the instantaneous voltage measurement is carried out using an oscilloscope. Ohm’s law can measure the current, and then the power is calculated by multiplying the voltage and the current.

The required power consumption can be obtained from state-of-the-art experimental studies on wireless data transfer. The reported power requirements are 109.76 µW [34], 900 µW [35], and 400 to 600 µW depending on the transmission frequency [36], and the RF CMOS transmitter with 300 µW [37]. Therefore, on average, a power generation of 400 µW will provide power for a self-powered sensor; thus, by typical 3.3V-output power storage, at least 120 µA current is needed.

**Figure 4:**
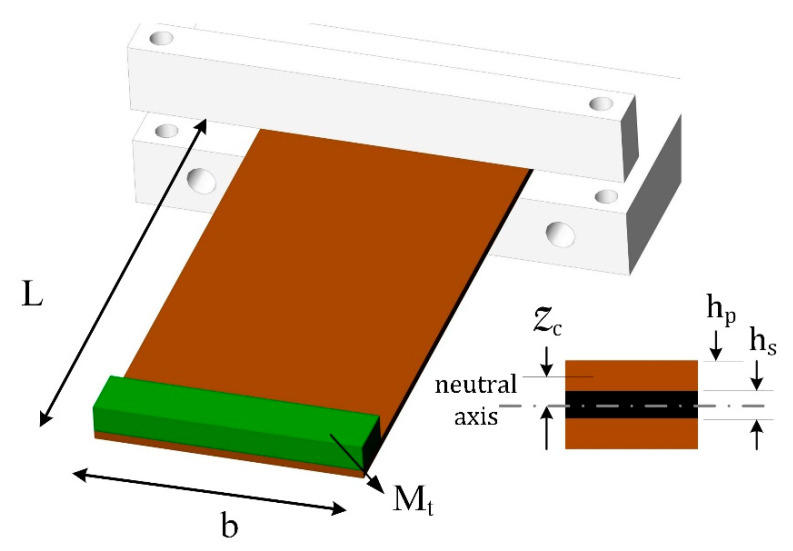
Piezoelectric bimorph with a tip mass.

**Figure 5:**
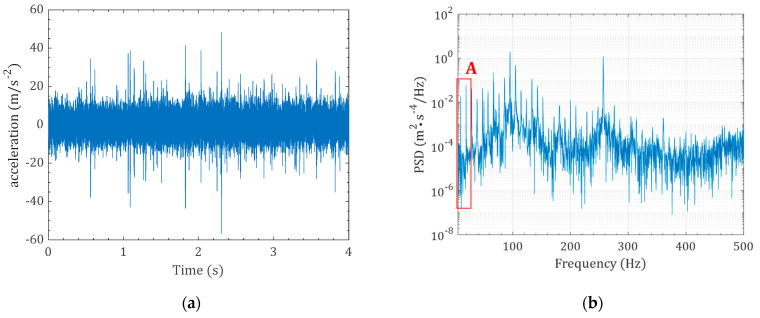

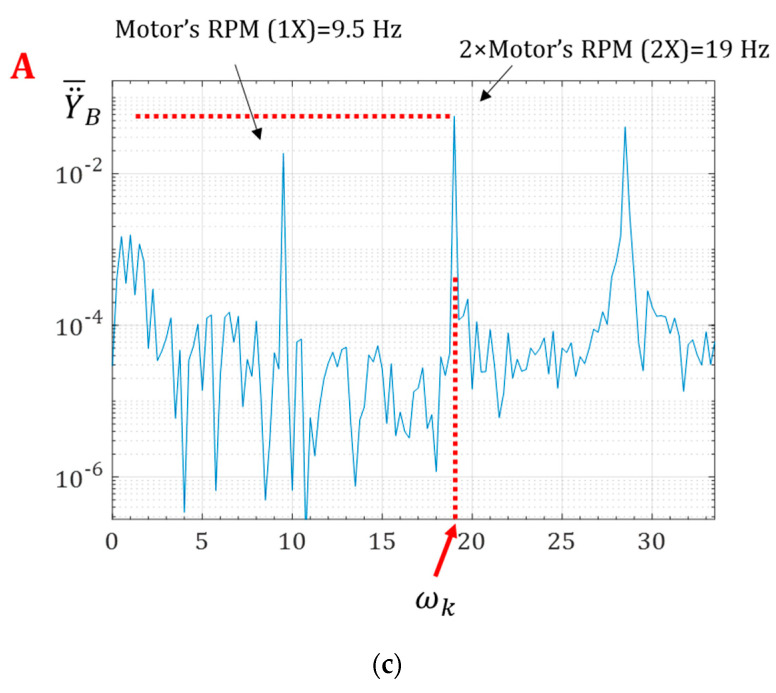
A stationary random vibration signal, (**a**) time domain signal ${\ddot{a}}_{B}\left(\omega \right)$ and (**b**) the $\Phi_{B}\left(\omega \right)$, and (**c**) zoomed-in view of the $\Phi_{B}\left(\omega \right)$ (A is the zoomed-in signal in the range of 0 to 35 Hz).

Figure 4 shows the piezoelectric generator (PG) with a rectangular tip mass. The inducing base excitation on the PG is a stationary random vibration denoted with ${\ddot{a}}_{B}\left(\omega \right)$, and the Fast Fourier Transform (FFT) is $\Phi_{B}\left(\omega \right)$. Figure 5a shows the time domain typical random vibration, and Figure 5b shows the FFT of the time domain signal. The FFT signal shows the dominant frequencies that are induced by the system operation. The rotor rotation speed, which is often known as the rotation speed, is one of the main dominant frequencies in the FFT. The rotation speed is called 1X frequency (the first rotation speed). The multipliers of rotation speed are discernible in the FFT signal, e.g., 2X (the first rotation speed times 2), 3X the first rotation speed times 3), etc. This rotation speed can be seen from the zoomed-in view of the FFT signal in Figure 5c.

**Figure 6:**
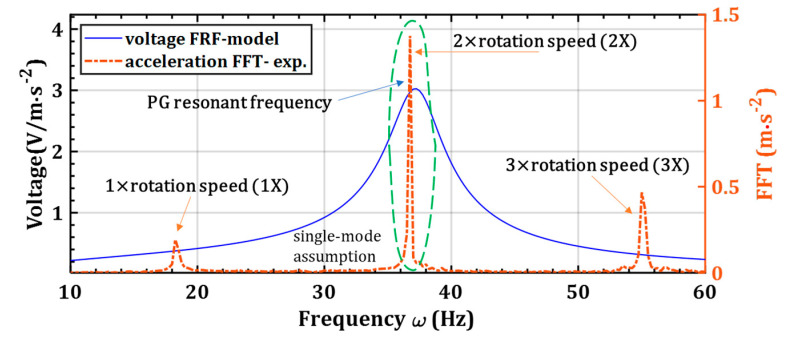
The PG’s voltage response and acceleration FFT, noting the narrow band for these signals, the voltage resonant frequency, and the 2X frequency of the acceleration FFT.

The PG has a narrow bandwidth, generating a maximum voltage from the signal within its natural frequency. On the other hand, the acceleration FFT is also narrowband. These narrowband signals for the piezoelectric voltage FRF and the vibration FFT are shown in Figure 6. Due to the narrowband signals, the acceleration in the dominant frequency around the PG’s natural frequency contributes most to the power generation. Thus, for simplicity, the single-mode harmonic excitation is considered.

The motor’s rotation speed ranges from 9.5 Hz to 23.0 Hz, while the harvester’s natural frequency with an 8.9-g tip mass is 33.2 Hz. There is a considerable gap between the natural frequency and the 1X motor’s rotation speed. Nevertheless, the second motor’s harmonic acceleration, e.g., 2X, lies within the harvester’s natural frequency, as shown in Figure 6. Therefore, the 2X frequency is considered as the exaction for the simulation model, e.g., $\omega_{k}$ (excitation frequency of the PG) is $2f_{r}$, where $f_{r}$ is the motor’s rotation speed.

It is considered that the input acceleration has a dominant frequency of $\omega_{k}$ with magnitude of ${\bar{\ddot{Y}}}_{B}$, and these parameters are estimated from the real-time experimental tests. From an analytical beam model [38], if the piezoelectric linear generator is excited by a harmonic load that is given by ${\ddot{Y}}_{B}\left(t\right)={\bar{\ddot{Y}}}_{B}\cos\left(\omega_{k}t\right)$ (${\bar{\ddot{Y}}}_{B}$ is the vibration acceleration in m/s^2^ and $\omega_{k}$ is the excitation frequency in rad/s), then the steady-state voltage output per acceleration (voltage transfer function) can be expressed by:

(4) $$H_{V\alpha }\left(\omega \right)=\frac{V_{\mathrm{steady}}}{{\bar{Y}}_{B}\omega_{k}^{2}}=\left|\frac{\frac{j\omega_{k}\Lambda \sigma }{\omega_{n}^{2}-\omega_{k}^{2}+j2\zeta_{i}\omega_{k}\omega_{n}}}{\frac{1}{R_{\mathrm{eff}}}+j\omega_{k}\frac{C_{P}}{2}+\frac{\omega_{k}\Lambda \Upsilon }{\omega_{n}^{2}-\omega_{k}^{2}+j2\zeta \omega_{k}\omega_{n}}}\right|$$

wherein $\omega_{n}$ and ζ are the natural frequency and damping coefficients, which are the pure mechanical vibration features of the PG, and σ, Λ, $C_{P}$, and Υ are the electromechanical coupling features of the PG. These parameters are given in Table 2.

As seen from Equation (4), any change in the excitation signal characteristics, either amplitude ${\bar{Y}}_{B}$ or frequency ω, varies the piezoelectric voltage output. Therefore, the piezoelectric voltage generation is damage sensitive, as demonstrated in Figure 2.

## 3. Experimental Setup for Measurement

**Figure 7:**
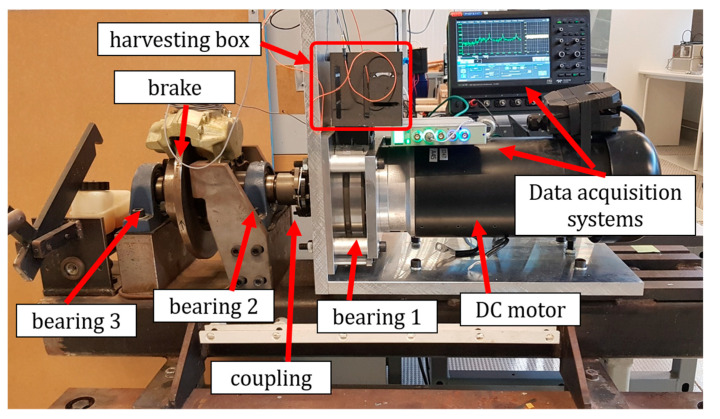
Experimental setup, the DC motor with an energy harvesting box attached to the main bearing, with data recording systems.

All the experimental tests were carried out on a V88.57 DC motor from Drive Systems, as shown in Figure 7. This DC motor was a 1.7 kW motor that consumed a maximum current of 85A with a 24 V voltage supply and could reach 1500 Rpm (≈25 Hz). The motor’s output shaft was connected to three bearings (numbered 1 to 3), a coupling, and a brake system. Figure 7 shows the DC motor setup, locations of the bearings, and the energy harvesting box. An oscilloscope was used to record the voltage output from the piezoelectric harvester. Besides, a B&K4517 accelerometer with a B&K3676 data acquisition system was employed for measuring the DC motor’s acceleration that was acting on the piezoelectric base.

**Figure 8:**
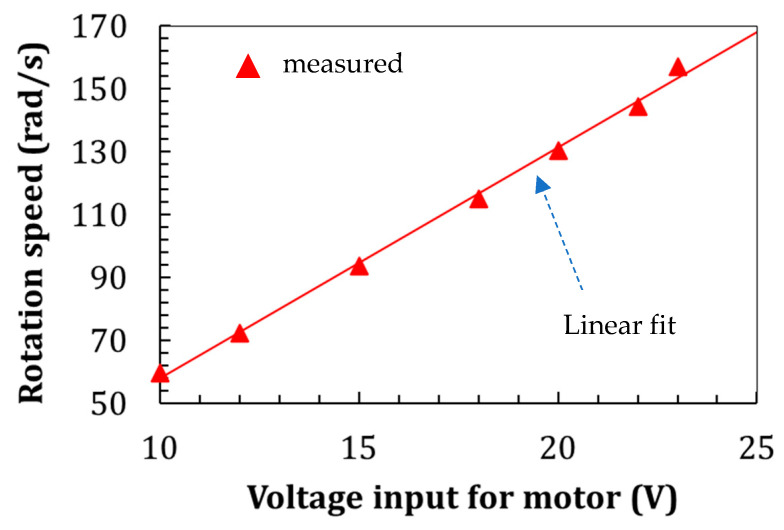
The motor’s supply voltage and rotation speed are linear relationships.

The motor rotational speed can be varied by changing the supply voltage. There is a linear relationship between the supply voltage and the rotational speed, as seen in Figure 8.

**Figure 9:**
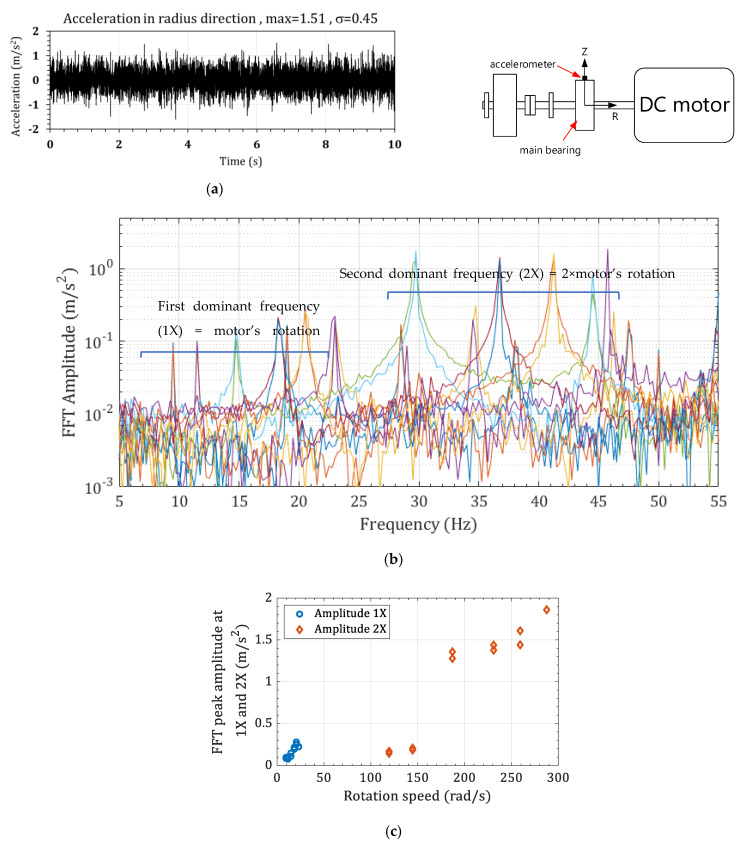
The acceleration on the main bearing in (**a**) R direction for 23 Hz rotation, (**b**) the FFT signals at different rotation speeds, and (**c**) the FFT acceleration peak at 1X and 2X rotation speeds.

Figure 9a shows a typical acceleration signal that is measured in the radius (R) direction. The Fast Fourier Transform (FFT) can convert this acceleration into a frequency-domain signal, see Figure 9b. The FFT represents a signal with a series of harmonic functions; therefore, the motor’s rotation speed and its higher multipliers exist in the FFT signal. In the FFT signal in the 5-to-55-Hz-span, two dominant frequencies can be seen, the first dominant frequency (1X) (the motor’s rotation speed) and the second dominant frequency (2X) (the second multiplier of the motor’s rotation speed). Accordingly, Figure 9b shows the FFT signals at all the rotation speeds in a frequency range containing the first (1X) and second rotation speeds (2X). The acceleration peaks for the 1X and 2X frequencies are shown in Figure 9c. The 2X’s peak amplitude is considerably higher than 1X’s, making the 2X frequency more appropriate for the piezoelectric harvester design.

## 4. Piezoelectric Generator Model Verification

**Figure 10:**
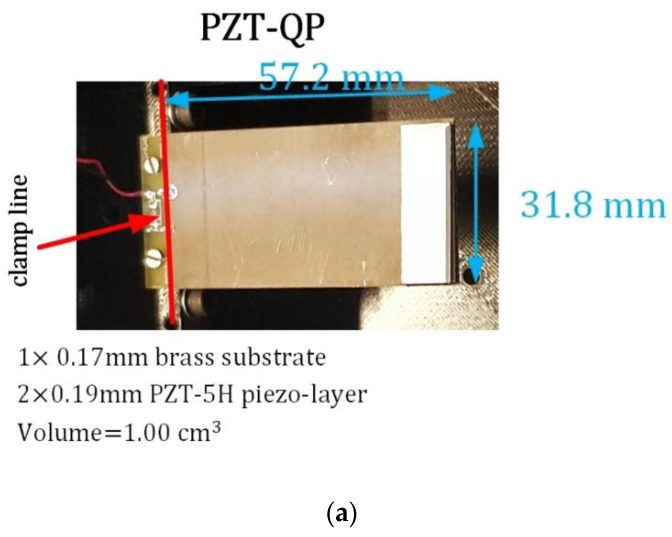

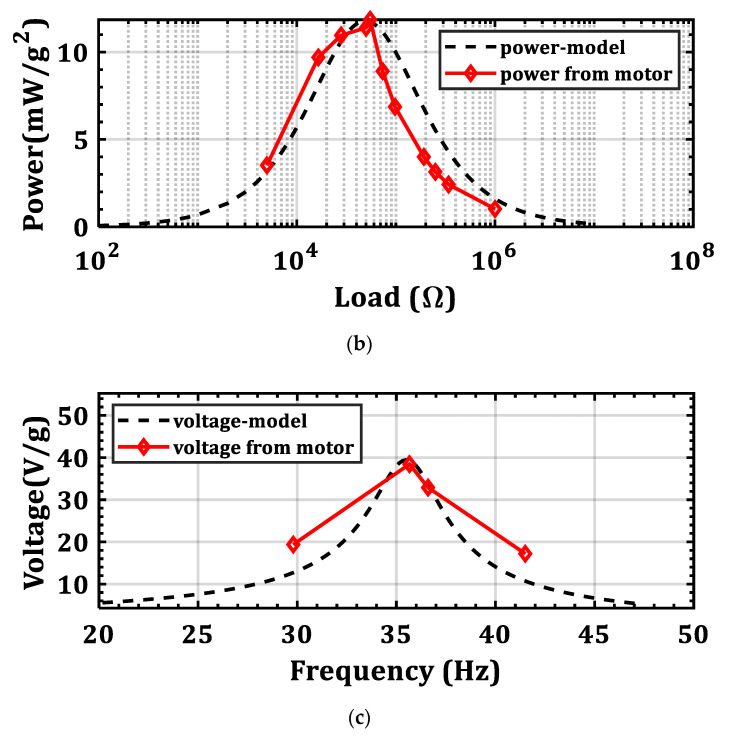
Experimental verification of the model, (**a**) PZT QuickPack model, (**b**) power versus electrical load at 2X-rotation-speed motor excitation, and (**c**) voltage versus frequency at $R_{l}=110\;k\Omega$ (g = 9.81 m/s^2^).

For model verification, a pre-wired bimorph from piezo.com [39] made from Piezoceramic PZT-5H, with an 8.9-g tip mass, served as the piezoelectric energy harvester, Figure 10a. This commercial bimorph is called PZT-QuickPack (PZT-QP) here. The output of the PG analytical model was compared with the piezoelectric voltage output under the DC motor working conditions. Young’s modulus for piezoelectric and substrate were 66.9 and 111.4 GPa, and the density for piezoelectric and substrate were 7750 and 8300 kg/m^3^. The damping coefficient was derived from the bandwidth analysis as 4.0%. The relative dielectric coefficient and piezoelectric constant *d*_31_ were 3800 and −320 × 10^−12^ C/N. The piezoelectric layers in this commercial sample were in parallel connection.

Table 3 compares the first natural frequency with and without the tip mass. The present model estimates the non-tip mass natural frequency at 79.3 Hz, which agrees with the manufacturer’s datasheet [39] and the finite element method [40]. Adding an 8.9-g tip mass reduces the natural frequency to 33.6 Hz, similar to the identified finite element result. Therefore, the present model simulates the mechanical PG behavior accurately. 

Various electrical loads and rotation speeds were examined, and the power and voltage outputs are plotted in Figure 10b,c, respectively. In Figure 10b, the overall trend in the power-load graph between the experiment and the model agrees that the voltage increases from small loads to the maximum power and decreases in the high loads. The agreement is satisfactory, especially 10^4^ to 10^5^ Ω. Moreover, Figure 10c shows the voltage versus the frequency. The agreement between the model and the experiment is reasonable. However, there are differences between the model and experiments for off-resonant excitations. This change can be due to the harmonic assumption while the DC motor has stochastic vibration. Overall, the trend for power and voltage estimation using this simple beam model is satisfactory.

## 5. Results and Discussions

The results section presents the energy harvesting aspects of the experimental DC motor, fault effects on the piezoelectric transducer, and the fault detection results

### 5.1. Energy Harvesting from the Motor’s Main Bearing

**Figure 11:**
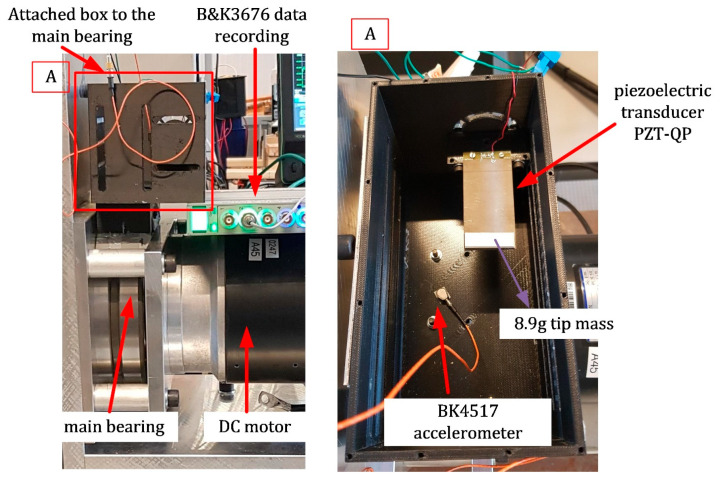
The pre-wired bimorph (PZT-QP) for the energy harvesting from the DC motor at different rotation speeds. A is the energy harvesting box in detail.

This subsection investigates the optimum power output from the energy harvesting box under the DC motor excitation. While the energy harvesting box can accommodate more than one PG, as the initial investigation, only one PG is attached to the harvesting box. Figure 11 shows the PZT-QP sample and its location on the main bearing.

**Figure 12:**
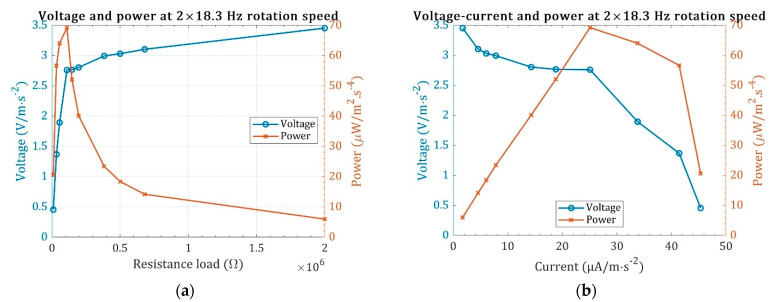
Finding the optimum load for the highest power generation. (**a**) The voltage and power versus the resistance load, and (**b**) the voltage-current and power-current curves.

The optimum PG power generation is obtained while the optimum load and frequency matching are implemented. Though the optimum load is frequency-dependent, the resonant excitation is often considered for designing a resonator. Similarly, the PG power generation is studied under the resonance excitation from the motor vibration, which occurs at approximately 2 × 18.3 Hz. The voltage and power versus the resistance load were measured and are plotted in Figure 12a,b under 12 × 8.3-Hz stochastic motor vibration. Typically, in single harmonic excitation, the voltage increases with increasing the electrical load resistance [16], same as here with stochastic vibration. Increasing the resistance load increases the voltage output, from 0.45 V/m·s^−2^ at 10 kΩ to 3.45 V/m·s^−2^ at 2 MΩ. The current, on the other hand, reduces by increasing the resistance. Thus, the power, the product of the voltage and current, has an optimum value at a specific load resistance. Specifically, the power output peak with the R_opt_ = 110 kΩ is maximum with 69.21 µW/m^2^·s^−4^ power at 25.08 µA/m·s^−2^ current and 2.76 V/m·s^−2^ voltage.

The motor’s rotation speed dictates the dominant frequencies in the vibration signal from the DC motor, which naturally affects the piezoelectric power output. From the theoretical model [18], if the dominant frequencies in the stochastic practical vibration signal are close to the harvester’s natural frequency, then the piezoelectric harvester will generate higher power. To test this hypothesis under a practical test, the effect of changing the motor’s rotation speed on the piezoelectric power generation was investigated. First, the rotation speed was varied, and the power output was recorded. 

**Figure 13:**
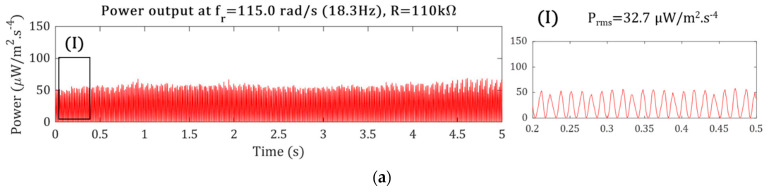

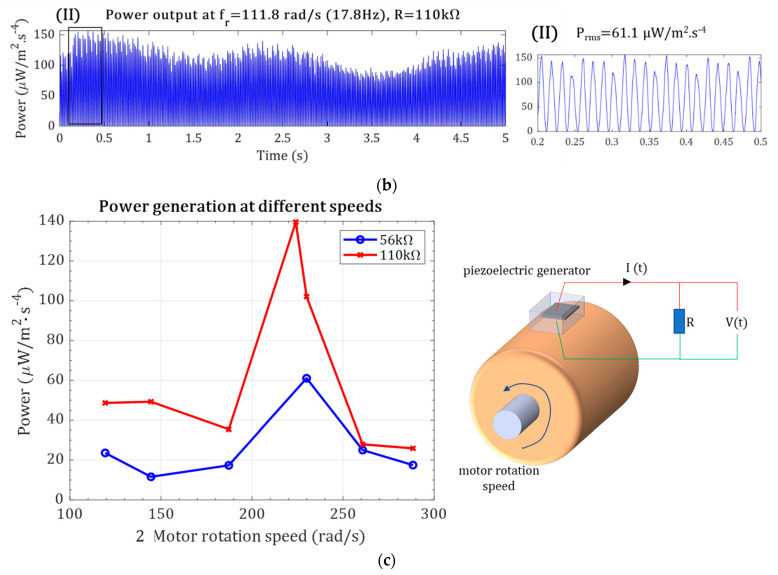
Normalized power per acceleration under (**a**) f_r_ = 18.3 Hz and R = 10 kΩ, (I) is the zoomed-in view for (**a**), (**b**) f_r_ = 17.8 Hz and R = 10 kΩ, (II) is the zoomed-in view for (**b**), and (**c**) different motor rotations with non-optimum (56 kΩ) and optimum-resistance loads (110 kΩ).

The power versus rotation speed is shown in Figure 13; the power at a specific frequency is three times greater than the power generation at the other frequencies. Therefore, the maximum power was 139.6 µW/m^2^·s^−4^ and obtained for the 2 × 17.8 Hz rotation speed. Moreover, if the rotation speed slightly increases to 2 × 18.3 Hz, a sharp drop occurs in the power generation. Therefore, the theoretical hypothesis is also proven by the practical stochastic vibration. Thus, it can be concluded that the power output is maximum when the dominant frequency of the vibration signal matches the harvester’s natural frequency. In addition, there is a strong dependency of the output power on the rotation frequency, proving that the narrow bandwidth also exists in the practical stochastic vibration case.

The power generation is highly rotation-speed-dependent and generates more significant power at the rotation frequency of 2 × 17.8 Hz than the 2 × 18.3 Hz rotation speed and reaches over 140 µW/m^2^·s^−4^, as shown in Figure 13. As a sign of resonance matching, the beating phenomena can be seen in the power output signal in Figure 13. A 1.0-Hz difference in the rotation speed causes an 84% power increase in the RMS of power output.

### 5.2. Toward a Self-Powered Online Condition Monitoring System

**Figure 14:**
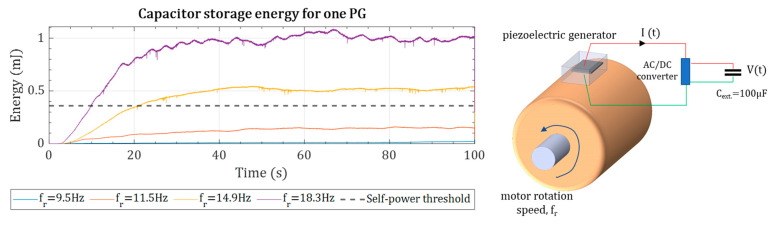
The stored energy for a 100 µF capacitor at different motor rotation speeds.

An energy of 360 µJ is needed for a fully autonomous wireless vibration [34]. For energy-storing investigation, a 100 µF capacitor is connected to the PG, and the stored energy at various motor rotation speeds is measured, as shown in Figure 14. As can be seen from Figure 14, at $\omega_{k}$ equals to 2 × 14.9 and 2 × 18.3 Hz, the stored energy crosses the threshold, while for low-rotation speeds, the stored energy does not reach the threshold in 100 s. For satisfying the autonomous condition monitoring, the power requirements shall be met at all the operating rotation speeds. Thus, in some rotation speeds, more than one PG is required.

The autonomous online condition monitoring system requires 400 µW power at 3.3 V and 120 µA. The piezoelectric energy harvesting box should provide this power and current at all the rotation speeds. For estimating the number of required PGs at each rotation frequency, an efficiency of 80% is considered for the AC/DC converter, DC/DC converter, and a microprocessor and storage. Assuming the same standard configuration for the PG, eight piezoelectric generators can achieve a fully self-powered wireless data transfer by eight piezoelectric generators, as shown in Table 4.

### 5.3. Fault Effects on the Piezoelectric Transducer as a Sensor

**Figure 15:**
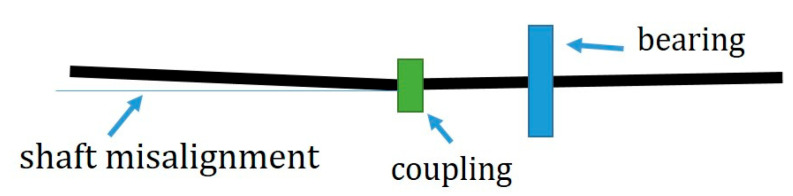
A 1.5% shifting of the shaft end creates a shaft-misalignment fault.

As an experimental study for assessing the DC motor’s health condition on the piezoelectric power generation, a shaft misalignment was created by deviating one shaft end 1.5% from the center. Figure 15 shows an exaggerated view of the shaft misalignment. 

**Figure 16:**
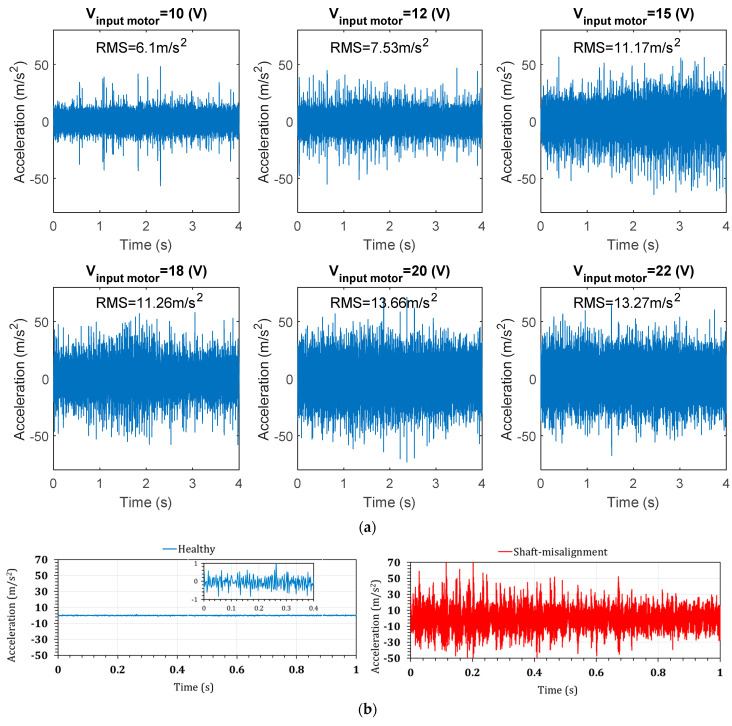
(**a**) The measured acceleration in the Z-direction for the shaft-misaligned DC motor, and (**b**) comparison of the acceleration in healthy and shaft-misalignment conditions.

As a result of the shaft misalignment, the DC motor’s acceleration increases considerably. Figure 16a shows that the acceleration ranges from 6.1 m·s^−2^ at V_input-motor_ = 10 V to 13.3 m·s^−2^ at V_input-motor_ = 22 V, which is substantially higher than the healthy-condition DC motor acceleration (see Figure 16b). As the input acceleration is changed due to the motor’s health condition, and the piezoelectric power generation depends on the input acceleration, the voltage generation is expected to change meaningfully.

**Figure 17:**
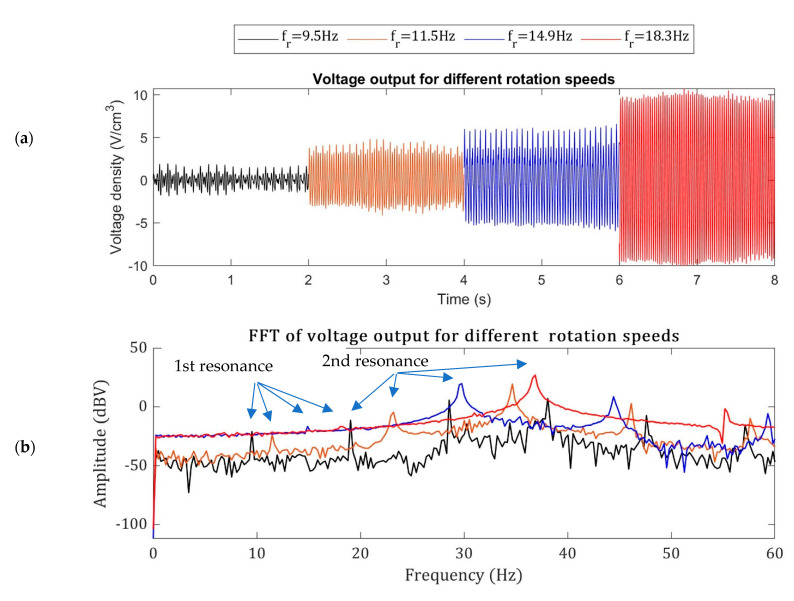
The open-circuit voltage output from the shaft-misaligned DC motor at different supply voltages. (**a**) The time-domain signals and (**b**) the FFT signals.

Figure 17 shows the open-circuit voltage output from the piezoelectric bimorph in the shaft-misalignment condition at different rotation speeds. As shown in Figure 17a, the time-domain voltage signals show that the voltage generation increases by increasing the rotation speed to 18.3 Hz. This trend was described in Figure 13, where the piezoelectric harvester generates the maximum power when there is a frequency match. The DC motor’s voltage output in the shaft-misaligned condition is 1.9 V for $f_{r}$ = 9.5 Hz and 10.2 V for $f_{r}$ = 18.3 Hz. The FFT of the voltage signals, shown in Figure 17b, gives the frequency content and the resonant peaks. The first and second resonances in the FFT plot are shown in Figure 17b.

**Figure 18:**
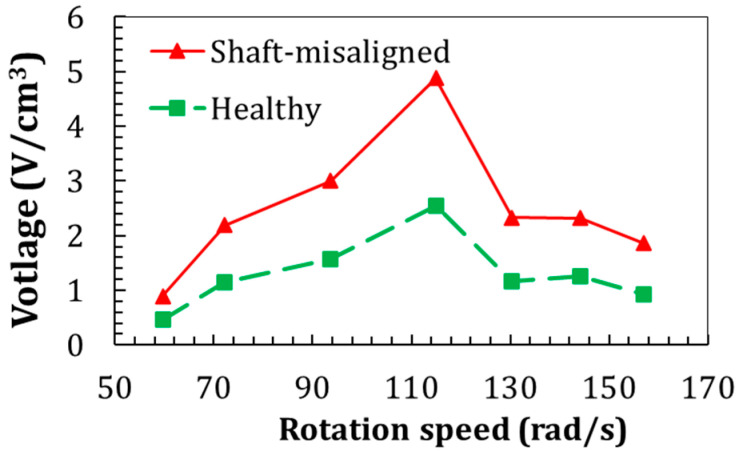
The comparison of voltage density from the DC motor at healthy and shaft-misaligned conditions.

As demonstrated in Figure 18, the DC motor’s voltage generation under the shaft-misaligned conditions is considerably higher than the healthy condition at all the tested rotation speeds. This increase is between 85% to over 100%, depending on the rotation speeds. This conclusion agrees with the numerical study results on a water pump [18], where the shaft-misaligned condition increases the voltage generation. Based on these piezoelectric voltage outputs, the damage index is evaluated.

### 5.4. Fault Detection

**Figure 19:**
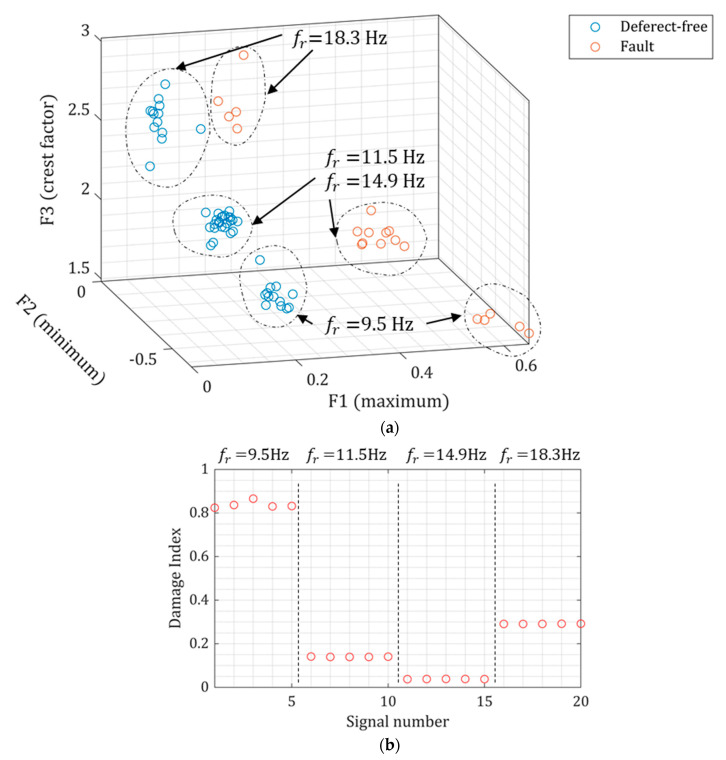
(**a**) Statistical features from the piezoelectric outputs at the defect-free and fault states, and (**b**) the damage index for the fault signals.

The fault detection is proposed using the unsupervised outlier method based on the MD function. The three statistical features (maximum, minimum, and crest factor) of the piezoelectric outputs at the defect-free and fault conditions are shown in Figure 19a. Even though the piezoelectric outputs are frequency normalized, the features are still frequency-dependent since the relationship between the piezoelectric output and frequency is nonlinear, see Equation (4). Thus, the dominant frequency in the Fourier Transform of the piezoelectric output is also considered the fourth feature. Nevertheless, the defect-free and fault states’ features are reasonably separated; therefore, the outlier method can be a suitable classifier. The damage index (DI) from the MD function is shown in Figure 19b. The damage indexes are clearly above zero, demonstrating a good separability from the defect-free state data.

The presented fault detection results are obtained from the measurements in a short period, while the long-term performance of such OCM systems needs further studies. Environmental and operational variables (EOVs) can hide the damage effects in the extracted features by changing the excitation amplitude or stiffness changes [41].

**Figure 20:**
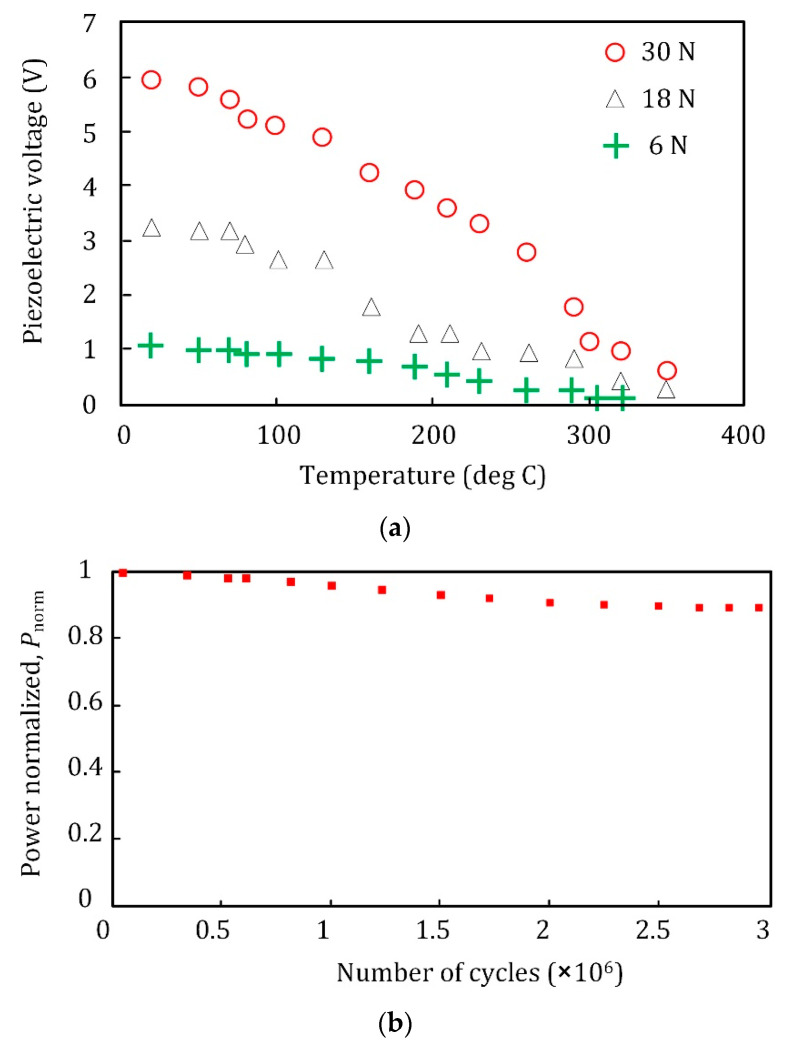
The effect of (**a**) temperature [42] and (**b**) long-term fatigue cycles [43] on the piezoelectric transducer output.

Fatigue behavior due to cyclic loads and temperature rise due to long-term machine operation can be two important EOVs. Figure 20a shows the temperature effect on the piezoelectric output for Piezoceramic PZT-5A [42], and Figure 20b shows the piezoelectric output versus the number of cycles for the Piezoceramic PZT-5H [43]. For our case study with ≤6 N excitation force, a temperature change from 20 °C to 100 °C reduces the piezoelectric output by 14.3%. Moreover, 3 × 10^6^ cycles reduce the piezoelectric power output by 11%. Thus, EOV influences the piezoelectric sensor performance, and their effects shall be considered. If the EOV effects are experimentally investigated, their effects can be explicitly included in the training model. The EOV factors can be a limitation for this type of sensor; however, their effects can be minimized by encapsulating the piezoelectric transducer, similar to the commercial sensors.

## 6. Conclusions

This paper investigates piezoelectric transducers’ autonomous online condition monitoring when the rotating machine works at a fully operational capacity. A DC motor is studied as the real-time showcase, and a model investigates the piezoelectric generator (PG) performance. The PG power output is investigated at different rotation speeds when it is connected to the main bearing for the self-powering aspect of wireless data transfer. Under the DC motor case, the optimal electrical load and the maximum generated power are measured, indicating a maximum and root mean square power of 13.43 mW/g^2^ and 5.9 mW/g^2^, where g = 9.81 m/s^2^. For the fault sensitivity, comments are made about the piezoelectric voltage output that are influenced by the damage. Moreover, experimentally, the PG voltage under defect-free and shaft-misaligned motor excitation is calculated, demonstrating a more than 200% voltage increase due to the shaft-misalignment. This conclusion opens the door to the autonomous online monitoring of rotating machines to detect faults that substantially change the vibration amplitude or frequency spectrum.

The current method has the limitation that a piezoelectric transducer can detect the mechanical faults that change the vibration level or frequency contents amplitude. Advanced fault detection techniques using machine learning techniques are proposed for future work for more advanced online condition monitoring systems. Moreover, the long-term performance and temperature effects are proposed for future works.

## Figures and Tables

**Table 1:** Effects of different faults on the vibration characteristics.

Fault Type	Frequency Shift (Hz)	Vibration Amplitude Change (%)
Bearing corrosion [7]	−2.16	100.4%
Separator damage [7]	+0.80	59.0%
Hot temperature [7]	+0.61	86.9%
Without lubricant [7]	1.85	66.1%
Shaft misalignment [28]	0.0	200.0%
Shaft looseness [28]	0.0	48.2%
Broken rotor bar [2]	±4.35	N/A
Bearing defect (hole in case) [2]	+120	N/A
Bent shaft [3]	0.0	351.0%

**Table 2:** Modal electromechanical parameters that are associated with vibration and electrical equations [38].

	Definition		Definition	
YI	2b3Yshs38+c¯11Ehp+hs23−hs38	σ	$m^{*}\int_{0}^{L}\phi \left(x\right)dx+M_{t}\phi \left(L\right)$	
P	e¯31b2hphs24−hp+hs22	Υ	$P\left({\left.\frac{d\phi \left(x\right)}{dx}\right|}_{x=L}\right)$	
$m^{*}$	$b\left(2h_{p}+h_{s}\right)$	Λ	$-\frac{{\bar{e}}_{31}bZ_{c}}{2}\left({\left.\frac{d\phi \left(x\right)}{dx}\right|}_{x=L}\right)$	
$\omega_{n}$	${\left(\lambda L\right)}^{2}\sqrt{\frac{YI}{m*L^{4}}}$	$C_{P}$	Series connection	$\frac{{\bar{\varepsilon }}_{33}bL}{2h_{p}}$
			Parallel connection	$\frac{{\bar{\varepsilon }}_{33}bL}{h_{p}}$
$\phi \left(x\right)$	$\chi \left[cosh\left(\lambda x\right)-cos\left(\lambda x\right)+\alpha_{i}\left(sinh\left(\lambda x\right)-sin\left(\lambda x\right)\right)\right]$	$R_{\mathrm{eff}}$	Series connection	$R_{L}$
			Parallel connection	$2R_{L}$

**Table 3:** The first natural frequency comparison between the present model, manufacturer’s datasheet, and the finite element.

Natural Frequency (Hz)	Datasheet [39]	Finite Element [40]	Present Model (from Table 2)
No tip mass	78.0	79.2	79.3
8.9-g tip mass	N/A	32.5	33.6

**Table 4:** The required number of PGs for autonomous wireless data transfer using the standard PG configuration.

*f_r_*, Rotation Speed (Hz)	9.5	11.5	14.9	17.83	18.3	20.75	22.95
Generated power with R = 110 kΩ, µW	99.0	256.5	401.3	1581.6	1029.2	238.3	236.5
Minimum required number of PGs	8	4	2	1	1	4	4
The number of required PGs	8						

## Data Availability

Not applicable.
